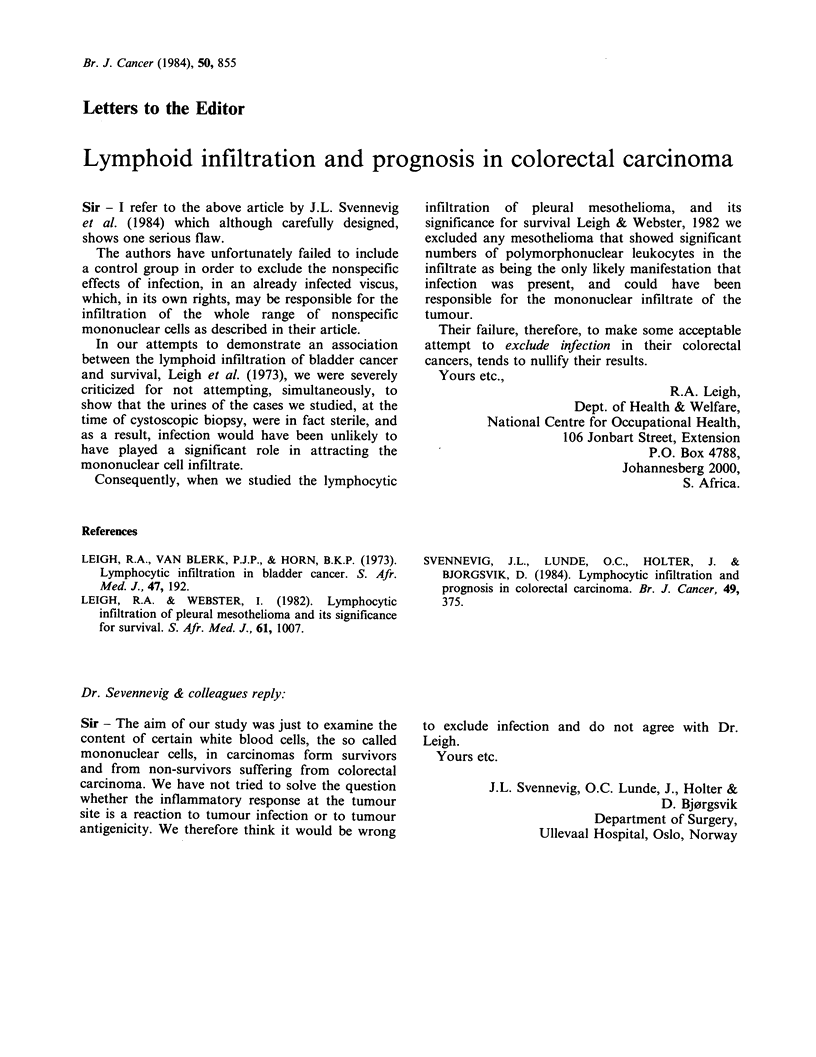# Lymphoid infiltration and prognosis in colorectal carcinoma

**Published:** 1984-12

**Authors:** R.A. Leigh


### Letters to the Editor

### Lymphoid infiltration and prognosis in colorectal carcinoma

**Sir** – I refer to the above article by J.L. Svennevig *et al.* (1984) which although carefully designed, shows one serious flaw.

The authors have unfortunately failed to include a control group in order to exclude the nonspecific effects of infection, in an already infected viscus, which, in its own rights, may be responsible for the infiltration of the whole range of nonspecific mononuclear cells as described in their article.

In our attempts to demonstrate an association between the lymphoid infiltration of bladder cancer and survival, Leigh *et al.* (1973), we were severely criticized for not attempting, simultaneously, to show that the urines of the cases we studied, at the time of cystoscopic biopsy, were in fact sterile, and as a result, infection would have been unlikely to have played a significant role in attracting the mononuclear cell infiltrate.

Consequently, when we studied the lymphocytic infiltration of pleural mesothelioma, and its significance for survival Leigh & Webster, 1982 we excluded any mesothelioma that showed significant numbers of polymorphonuclear leukocytes in the infiltrate as being the only likely manifestation that infection was present, and could have been responsible for the mononuclear infiltrate of the tumour.

Their failure, therefore, to make some acceptable attempt to *exclude infection* in their colorectal cancers, tends to nullify their results.

Yours etc.,

R.A. Leigh,
Dept. of Health & Welfare,
National Centre for Occupational Health,
106 Jonbart Street, Extension
P.O. Box 4788,
Johannesberg 2000,
S. Africa.

**References**

LEIGH, R.A., VAN BLERK, P.J.P., & HORN, B.K.P. (1973). Lymphocytic infiltration in bladder cancer. *S. Afr. Med. J.*, **47**, 192.

LEIGH, R.A. & WEBSTER, I. (1982). Lymphocytic infiltration of pleural mesothelioma and its significance for survival. *S. Afr. Med. J.*, **61**, 1007.

SVENNEVIG, J.L., LUNDE, O.C., HOLTER, J. & BJORGSVIK, D. (1984). Lymphocytic infiltration and prognosis in colorectal carcinoma. *Br. J. Cancer*, **49**, 375.